# Supplementary material for: The genome sequence of White-foot Bell moth, Epiblema foenella (Linnaeus, 1758)
Source: Wellcome Open Res. 2024 Nov 20;9:686. [Version 1] doi: 10.12688/wellcomeopenres.23364.1 (PMC11803198; doi:10.12688/wellcomeopenres.23364.1)
Supplement: Peer review history

# Reviewer report: Reviewer response for version 1

**DOI:** 10.21956/wellcomeopenres.25754.r114499

**Published:** 2024-12-23

**Authors:** Nicola J Nadeau

**Affiliations:** 1 The University of Sheffield, Sheffield, UK

There is sufficient background to the species. The methods are described clearly and fully. The data is accessible. The chromosome-level assembly appears to be of good quality based on the reported metrics.

 Although transcriptomic data has been generated and is included with the deposited data, annotation is not described in this paper (except for BUSCO) and the annotation does not yet appear to be available in Ensembl. This will add to the utility of the genome, once available.

Are sufficient details of methods and materials provided to allow replication by others?

Yes

Is the rationale for creating the dataset(s) clearly described?

Yes

Are the datasets clearly presented in a useable and accessible format?

Yes

Are the protocols appropriate and is the work technically sound?

Yes

Reviewer Expertise:

Butterfly evolutionary genetics/genomics

I confirm that I have read this submission and believe that I have an appropriate level of expertise to confirm that it is of an acceptable scientific standard.

# Reviewer report: Reviewer response for version 1

**DOI:** 10.21956/wellcomeopenres.25754.r114501

**Published:** 2024-12-16

**Authors:** Michal Rindoš

**Affiliations:** 1 University of South Bohemia, Ceske Budejovice, Czech Republic

The manuscript contains all the relevant information about the biology and distribution of the studied *Epiblema foenella*. Also, the material and methods are well written including all the details needed. The cited literature includes approximately the same sources as all the other Genome Notes. I have a question regarding the COI Barcode obtained from the specimen. Was it published on BOLD?

Are sufficient details of methods and materials provided to allow replication by others?

Yes

Is the rationale for creating the dataset(s) clearly described?

Yes

Are the datasets clearly presented in a useable and accessible format?

Yes

Are the protocols appropriate and is the work technically sound?

Yes

Reviewer Expertise:

Biodiversity and systematics of Lepidoptera

I confirm that I have read this submission and believe that I have an appropriate level of expertise to confirm that it is of an acceptable scientific standard.
